# Progress towards malaria elimination: insights from Cambodia's mobile malaria workers during the COVID-19 pandemic

**DOI:** 10.1093/inthealth/ihaf080

**Published:** 2025-08-07

**Authors:** Emma K Manning, Rekol Huy, Sovannaroth Siv, Po Ly, James K Tibenderana, Lieven Vernaeve

**Affiliations:** Malaria Consortium, 244-254 Cambridge Heath Road, London E2 9DA, UK; National Center for Parasitology, Entomology and Malaria Control, #477 Be tong St(Corner St 92), Trapaing Svay village, Sangkat Kork Kleang, Khan Sensok, Phnom Penh 12101, Cambodia; National Center for Parasitology, Entomology and Malaria Control, #477 Be tong St(Corner St 92), Trapaing Svay village, Sangkat Kork Kleang, Khan Sensok, Phnom Penh 12101, Cambodia; National Center for Parasitology, Entomology and Malaria Control, #477 Be tong St(Corner St 92), Trapaing Svay village, Sangkat Kork Kleang, Khan Sensok, Phnom Penh 12101, Cambodia; Malaria Consortium, 244-254 Cambridge Heath Road, London E2 9DA, UK; Malaria Consortium, Phnom Penh Center, Street Sothearos, Tonle Basac, Chamkarmorn, Building “H”, 1st Floor, Room No. 192, Phnom Penh 12300, Cambodia

**Keywords:** Cambodia, COVID-19, malaria, mobile malaria workers, pandemic response

## Abstract

Significant progress has been made in Cambodia towards malaria control and the goal of achieving elimination of all species by 2025. These efforts require constant vigilance and agility in malaria programming to maintain forward momentum. During the COVID-19 pandemic, these achievements were threatened by restrictive pandemic control measures, necessitating swift action from the National Center for Parasitology, Entomology and Malaria Control and partners to safeguard critical malaria services, primarily case detection and treatment by mobile malaria workers (MMWs) among hard-to-reach populations. As malaria cases have declined in Cambodia, infections are increasingly focused among remote populations further from primary healthcare services. Thus, the greatest threat to malaria control during the pandemic consisted of the logistical and communication challenges faced by MMWs travelling to remote regions while movement was restricted. Through locally tailored implementation of these services and close communication with health authorities, Malaria Consortium's MMWs were able to minimise disruption of Cambodia's malaria control programme while ensuring staff and beneficiaries were protected.

## Introduction

Cambodia has achieved notable success in controlling malaria since implementing the National Strategic Plan for Malaria Elimination in 2011, which set the goal of attaining malaria elimination by the end of 2025. From 2003 to 2019, malaria incidence declined by 76% and no malaria deaths have been recorded in the country since 2018. Reporting only 355 cases in 2024, the country is on track to achieve malaria-free certification from the WHO by 2029.^1^ This progress has required vigilance to sustain the effectiveness of malaria control strategies in the face of emerging threats including artemisinin resistance and the coronavirus disease 2019 (COVID-19) pandemic. Although the first case of COVID-19 in Cambodia was recorded on 27 January 2020, restrictions to limit disease spread were quickly put in place and case numbers remained low until >1 y later. During the pandemic, Malaria Consortium worked with the National Center for Parasitology, Entomology and Malaria Control (CNM) to maintain mobile malaria services in remote, mobile and migrant populations in six provinces along Cambodia's international border with Laos, Thailand and Vietnam. In this retrospective review, we reflect on the lessons learned from the activities of mobile malaria workers (MMWs) throughout the pandemic that highlight the importance of Cambodia's unique adaptive approach to malaria control in forested and remote areas. The reflections in this case study are limited to the specific experiences of our organisation in contributing to the Royal Government of Cambodia's objective of malaria elimination.

## Mobile malaria workers: maintaining malaria services

### Malaria services in high-risk regions

At the onset of the pandemic, many Cambodians working across the border in Thailand returned home. To limit the number of imported cases and prevent the transmission of COVID-19 throughout their home provinces, health authorities established camps along the border for migrants to quarantine for 2–3 wk. Initially, all non-governmental organisations were instructed to cease operations in border areas while the camps were in place, which would have prevented the delivery of malaria services to the surrounding communities and limited activities throughout the border provinces. As Cambodia approaches malaria elimination, prevention, early case detection and immediate treatment among remote and mobile populations in these regions are vital. Discontinuation of interventions could have had a detrimental effect on malaria burden in these regions as it would likely have resulted in an increase of malaria cases, placing further strain on health systems. Through a long-established positive relationship with the local health authorities and collaboration to adapt the specific community-based programme delivery in remote areas, partners successfully advocated to continue the operations of MMWs at mobile malaria posts and during outreach activities, throughout the pandemic.^2^

### Local staff and locally recruited MMWs

While Malaria Consortium's activities in Cambodia are supported from the Phnom Penh office. Field officers are based in six provinces providing essential support and supervision for MMWs, based on national guidelines set out by the national malaria programme (CNM).^3^ MMWs are locally recruited, are often forest goers themselves, migrants or an indigenous population, and are integrated into the national malaria programme community network to provide early diagnosis and treatment of malaria. While village malaria workers (VMWs) traditionally offer passive case detection in fixed locations at village level, MMWs actively seek out infections, targeting high-risk populations with less access to health centres or village-based care.^4,5^ Supported by technical input and expertise from the CNM, MMWs deliver essential malaria services to remote and hard-to-reach communities.^3^

### Procurement and delivery of commodities

Pandemic control measures restricted movement between provinces, posing the greatest challenge to continued implementation of malaria services in hard-to-reach locations. Logistically, these restrictions hindered procurement and delivery of key malaria commodities. For example, delays interrupted the distribution of hammock nets used by forest workers to protect them while they are sleeping outside. The impact of these restrictions was minimal due to the preparedness of health centres with well-maintained stock levels of vital malaria commodities for diagnosis and treatments prior to the pandemic. Rather than having to focus on procurement, health centres were able to quickly adapt their method of distributing commodities to VMWs and MMWs to enable continued delivery within pandemic regulations. Health centres maintained monthly meetings for distribution, but instead of group meetings, VMWs and MMWs collected the materials one by one, staggered throughout the day. Stock of commodities, including rapid diagnostic tests (RDTs) and drugs used to treat malaria, was sufficient to supply VMWs and MMWs until restrictions were eased.

### Adapting communication and services delivery

Effective communication between health centres, programme staff and MMWs was essential to maintain malaria services. Lockdowns and travel restrictions impeded in-person supportive supervision by the Phnom Penh-based team to staff throughout the province, which typically occurred during monthly meetings and field visits. Transitioning communications to online platforms was ineffective due to limited connectivity in the northern provinces of Cambodia. To maintain communication, teams continued to meet in person where possible with strict infection prevention and control measures in place. During meetings, windows were opened, participants wore masks and social distancing was adhered to. Where in-person meetings were not possible and to reduce the number of meetings, province-based staff supported MMWs in remote areas over the phone.

Community-based programme delivery was also adapted. Prior to the pandemic, MMWs often gathered communities together for health education and promotion activities and organised community dialogues on health-seeking behaviour. Due to guidelines preventing large gatherings, these community dialogues were suspended. Instead, MMWs delivered health education to each household individually. Despite the door-to-door method being more time-consuming, there was no evidence of a decrease in testing or delivery of services. Data reporting meetings that typically occurred in-person during MMWs’ monthly visits to health centres were modified to reduce contact. Coinciding with malaria commodity resupply, MMWs staggered their visits to health centres and held individual reporting meetings outside while observing social distancing rules. No decrease in reporting occurred during this period without group data-reporting meetings.

## Learning and recommendations

### Resilience and adaptability of Cambodia's MMWs

The decentralised organisational structure and local recruitment of MMWs limited the impact of lockdowns and travel restrictions, strengthening our ability to provide continuous malaria services throughout the pandemic. All MMWs for malaria programmes in rural Cambodia are recruited by their communities and receive supervision, monitoring and materials from province-based staff. These local MMWs are recruited because they know the community, the movements of people within and speak the local languages, making them best suited to reach mobile and migrant populations in hard-to-reach locations. Initial lockdowns in Cambodia were confined to Phnom Penh, which prevented travel from the city to rural provinces but allowed our staff and MMWs throughout the six provinces to continue to deliver malaria services in rural areas without disruption. Although the staff and some MMWs faced challenges travelling between districts and provinces, often undergoing rigorous COVID-19 testing when crossing a border, their adaptability ensured that malaria services still reached populations that were otherwise inaccessible to health centres. The existing framework of the VMW and MMW programmes overseen by the CNM and the strong community relationships established by MMWs enabled a successful response to COVID-19 by being highly adaptable and agile.

### Existing infection, prevention and control practices

Cambodia, and the rest of Southeast Asia, has experience in dealing with novel respiratory diseases, such as avian flu, which are common in the area. As a result, some of the infection prevention and control measures implemented globally for COVID-19 were an existing feature in malaria control programmes in Cambodia. MMWs were already supplied with gloves and masks to protect them when performing RDTs and with hand sanitisers for when there is no access to handwashing facilities. These existing measures were an advantage in the early stages of the pandemic. While the rest of the world was procuring personal protective equipment (PPE), the malaria programmes in Cambodia had pre-existing stock and communities were accustomed to health workers wearing masks and gloves and using hand gel. Communities were not frightened by the presence of masks and gloves, which enabled the continued provision of services without a decrease in uptake. This, combined with the community trust earned by MMWs, encouraged sustained health-seeking behaviour at a time when, in many other countries, populations became hesitant to seek care due to the uncertainty around health guidelines.

### Looking forward

As Cambodia approaches malaria elimination, maintaining malaria services throughout the pandemic among hard-to-reach populations and remote communities was essential to prevent re-establishment of the disease, especially imported cases among Cambodians returning from working in Thailand. MMWs played a unique role in providing care to high-risk populations during the COVID-19 pandemic and were equipped to do so based on the existing infrastructure and CNM guidelines. These successes should be shared with other countries and work should be done to identify how these practices could be applied in different contexts. For example, national malaria programmes and funding agencies should prioritise building and maintaining supply stocks throughout rural areas and organisations delivering services should provide PPE as a standard measure to all field-based staff.

## Data Availability

The data underlying this article will be shared on reasonable request to the corresponding author.
